# Limit of blank and limit of detection of *Plasmodium falciparum* thick blood smear microscopy in a routine setting in Central Africa

**DOI:** 10.1186/1475-2875-13-234

**Published:** 2014-06-14

**Authors:** Fanny Joanny, Sascha JZ Löhr, Thomas Engleitner, Bertrand Lell, Benjamin Mordmüller

**Affiliations:** 1 Institut für Tropenmedizin, Eberhard Karls Universität Tübingen, Wilhelmstraße 27, 72074 Tübingen, Germany; 2 Centre de Recherches Médicales de Lambaréné (CERMEL), BP 118 Lambaréné, Gabon

**Keywords:** Thick blood smear, Microscopy, Diagnostics, Limit of blank, Limit of detection, Quality control

## Abstract

**Background:**

Proper malaria diagnosis depends on the detection of asexual forms of *Plasmodium spp.* in the blood. Thick blood smear microscopy is the accepted gold standard of malaria diagnosis and is widely implemented. Surprisingly, diagnostic performance of this method is not well investigated and many clinicians in African routine settings base treatment decisions independent of microscopy results. This leads to overtreatment and poor management of other febrile diseases. Implementation of quality control programmes is recommended, but requires sustained funding, external logistic support and constant training and supervision of the staff. This study describes an easily applicable method to assess the performance of thick blood smear microscopy by determining the limit of blank and limit of detection. These two values are representative of the diagnostic quality and allow the correct discrimination between positive and negative samples.

**Methods:**

Standard-conform methodology was applied and adapted to determine the limit of blank and the limit of detection of two thick blood smear microscopy methods (WHO and Lambaréné method) in a research centre in Lambaréné, Gabon. Duplicates of negative and low parasitaemia thick blood smears were read by several microscopists. The mean and standard deviation of the results were used to calculate the limit of blank and subsequently the limit of detection.

**Results:**

The limit of blank was 0 parasites/μL for both methods. The limit of detection was 62 and 88 parasites/μL for the Lambaréné and WHO method, respectively.

**Conclusion:**

With a simple, back-of-the-envelope calculation, the performance of two malaria microscopy methods can be measured. These results are specific for each diagnostic unit and cannot be generalized but implementation of a system to control microscopy performance can improve confidence in parasitological results and thereby strengthen malaria control.

## Background

Despite a significant reduction in the number of cases since 2000, malaria was still responsible for approximately one million deaths and 219 million cases in 2010, the majority in sub-Saharan Africa [1,2]. Better diagnosis and treatment is a cornerstone for malaria control and future elimination campaigns. Inaccurate diagnosis is problematic for the patient who receives either unnecessary or incorrect treatment with its risk of side effects and potential complications or prolonged disease episodes due to other underlying pathologies. In addition, patients may develop mistrust in the health care system when misdiagnosis leads to wrong treatment decisions [3]. Moreover, inaccurate treatment can exert drug pressure on parasite populations and leads to development of resistance [4]. Therefore, the World Health Organization (WHO) recommends that every case of malaria should be confirmed before starting treatment [1]. Since the development of Giemsa stain in 1904 by Gustav Giemsa [5], microscopy of Giemsa-stained thick blood smear (TBS) has been the gold standard for malaria diagnosis. In 2010, an estimated 165 million of patients were tested by microscopic examination [6]. TBS is comparatively cheap (0.10-0.40 US$), can have excellent sensitivity and allows discrimination between plasmodial species as well as other blood-dwelling parasites, such as microfilaria [3]. With minor modifications it can be used to quantify parasite density and haemozoin content for estimation of whole body parasite burden [7-9]. However, TBS depends on well-maintained microscopes of good quality and experienced microscopists, conditions that are difficult to achieve in many endemic settings. Lack of confidence in diagnostic performance of TBS in endemic areas is likely to be the source of the common clinical practice to treat with anti-malarials irrespective of parasitological results [10-12]. External quality assessment may improve the situation but is expensive, requires additional logistics and needs to be sustained to lead to improvement when baseline performance is poor [13]. An alternative strategy is implementation of internal procedures to assess diagnostic performance that can be communicated to clinicians and external parties, provides training and self-assessment and is easy to implement. In this study standard laboratory methodology was applied to assess diagnostic performance of TBS microscopy in a representative African research centre and guidance for implementation is given.

A particularly suitable measure of diagnostic performance for TBS microscopy by a group of microscopist is the estimation of the limit of blank (LOB) and the limit of detection (LOD). LOB corresponds to the highest parasite count that is likely to be observed (with a pre-specified probability) for a blank sample meaning that if a sample has a parasitaemia below this value, it can be considered as negative with a stated probability. In contrast, LOD is the lowest parasite count in a sample which can be detected with a pre-specified probability, although perhaps not quantified as an exact value [14]. It means that a TBS with a parasitaemia above this value has a stated probability to be truly positive. These two values allow a reliable discrimination between negative and positive slides and are informative on the quality of readings. The LOB and LOD of TBS were evaluated using case-adapted “The Clinical and Laboratory Standards Institute” (CLSI/NCCLS) guidelines [14] for the WHO and the Lambaréné TBS method. The Lambaréné method is a TBS modification that allows accurate parasite quantification because a defined volume of blood is spread over a defined area. It is routinely used in Lambaréné and other centres throughout Africa and Europe [8]. Although there are several ways to calculate LOB and LOD, the CLSI guidelines have been chosen notably because they are specifically designed for methods reporting “zero” or positive only values. The method described here is easily applicable and allows measurement of diagnostic performance with very little extra logistics, effort and material.

## Methods

### Study site and volunteers

The study was performed at the *Centre de Recherches Médicales de Lambaréné (CERMEL)* in Gabon. It received approval by the regional ethics committee (CERIL) and followed the principles of the Declaration of Helsinki (6^th^ revision). Following informed consent, blood samples were obtained from five adult malaria patients and five European volunteers recently arrived in Lambaréné, who had no history of malaria.

### Preparation of thick blood smear

**Figure 1:**
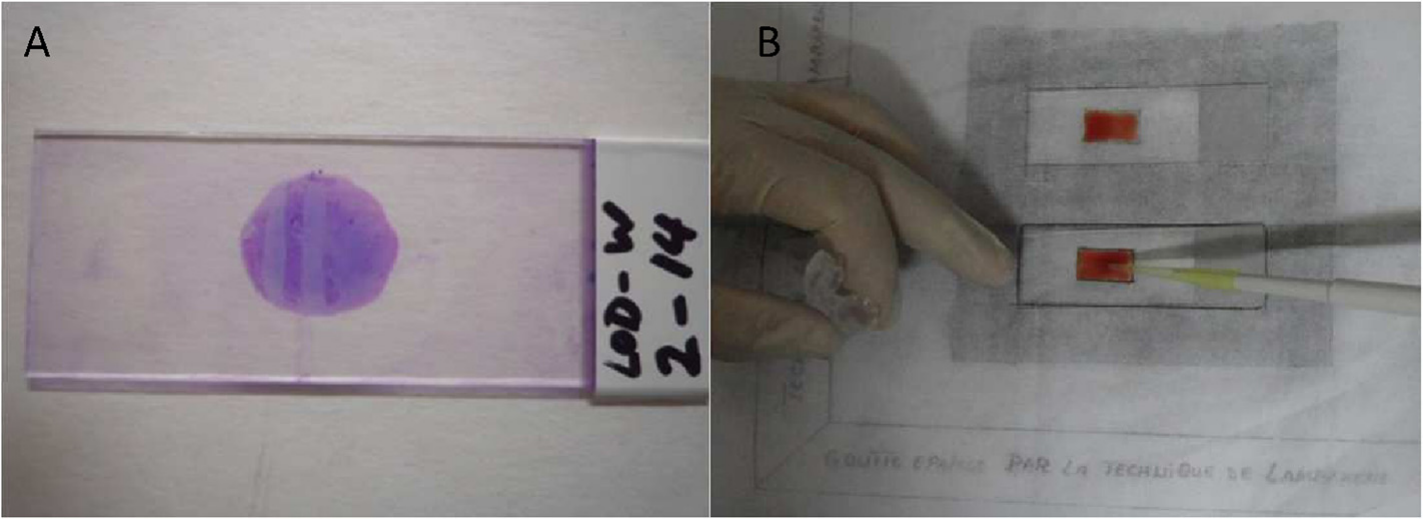
**Preparation of TBS. A)** TBS prepared according to the WHO method and stained with Giemsa; **B)** Preparation of a TBS by the Lambaréné method.

For the WHO method, 10 μL of EDTA blood was distributed on a circular area of a microscope slide (Figure 1). The slide was dried and stained for 20 minutes in a 20% Giemsa solution (Giemsa R-Solution, Merck, Darmstadt; Titrisol Puffer pH 7.2, Merck, Darmstadt). Four Giemsa preparations were used to have a better representation of laboratory routine. The slide was rinsed in tap water, dried and 100 high power fields (HPF) were observed under a 100x immersion oil objective (Nikon Eclipse E200, Tokyo, Japan or Olympus CH 30, Tokyo, Japan). The number of parasites (N_Parasites_) as well as the number of leucocytes (N_Leucocytes_) was counted and the parasitaemia was calculated using the measured or assumed (8,000 cells/μL) leucocytes count as reference:

$$\frac{\text{Parasites}}{\mu L}=\frac{N_{\text{Parasites}}\cdot \text{Leucocytes}/\mu L}{N_{\text{Leucocytes}}}$$

For the Lambaréné method, 10 μL of blood was evenly distributed over an area of 10x18mm, dried and stained as for the WHO method. The contour of the 10x18mm rectangle was drawn on a piece of paper placed below the slide before spreading the sample (Figure 1). The number of parasites as well as the number of read HPFs was counted (at least 100) and parasitaemia per microlitre was calculated by a multiplication factor that depends on the magnification and the area of the microscopic field and represents the number of HPFs to be read for the examination of 1 μL of blood and can either be derived from the optical specifications of the microscope or, as the preferred option, measured physically.

The two microscopes used in Lambaréné have a microscope factor of 560 and 800 and parasitaemia is calculated as follows:

$$\frac{\text{Parasites}}{\mu L}=\frac{N_{\text{Parasites}}}{N_{\text{HPF}}}\cdot \text{Microscope}\;\text{Factor}$$

For each slide, an ID number was given as follows: LOD-X-Y-Z (LOD: name of the study, X: method used [W for WHO or L for Lambaréné], Y: slide number and Z: number of the Giemsa coloration used [1 to 4]). To prepare the low parasitaemia slides, parasitaemia of each participant was determined and diluted with 0 + malaria negative blood to the required parasitaemia (10–100 parasites/μL).

### Randomization and blinding of the TBS

To avoid bias, TBS were randomized and blinded. To create a random sequence, a third person, independent from the experiment sorted low-parasitaemia and negative slides in random order. After this, each slide was given a new code on a non-transparent sticker hiding the ID number. The code consisted of the name of the study (LOD), the method used (L for Lambaréné, W for WHO), the sequence number and the rank within the sequence (e.g. LOD_L_2_4). After one random sequence was read by all the microscopists, a new random sequence was generated and blinded. As a consequence, each slide was read independently several times by all microscopists.

### Determination of the limit of blank (LOB)

**Figure 2:**
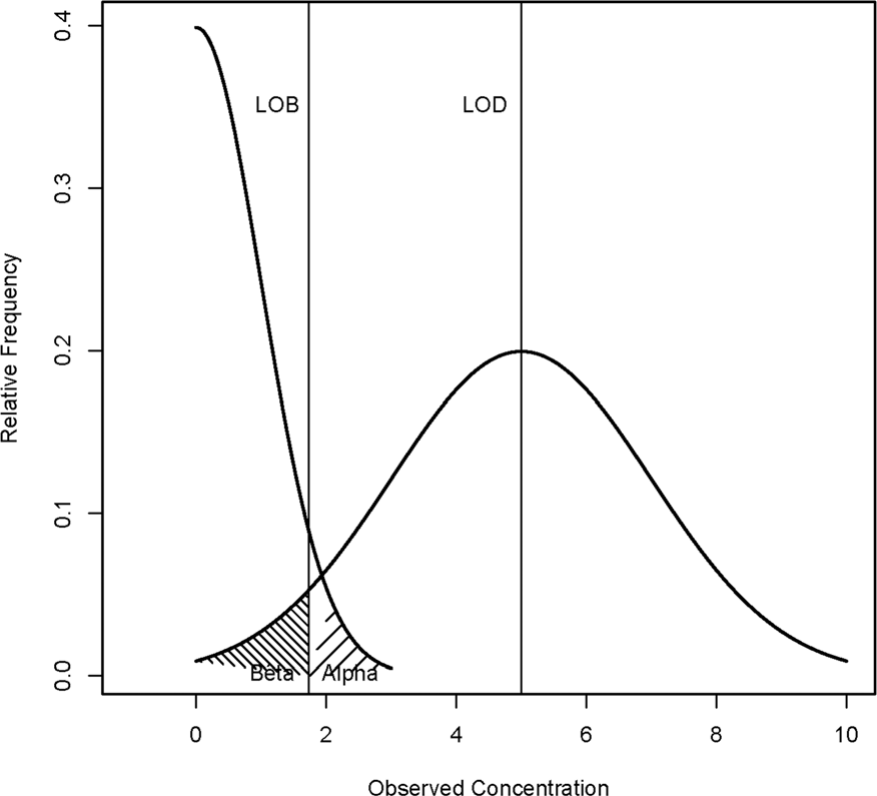
**Example for the distribution of a negative sample and one hypothetical low-level sample whose concentration is equal to the LOD.** In that case 95% (100%-β) of the measurements exceed the LOB. On the other hand, 95% of the measurements of the negative sample (100%-α) are below the LOB. Adapted from [14].

The LOB was estimated by reading replicates of negative slides and calculating the mean and the standard deviation from the results. It corresponds to a pre-defined percentile of the parasitaemia distribution of negative samples *LoB* = *Pct*_*B*(*100* - *α*)_ (Figure 2). Alpha (α) is the type I error and corresponds to the probability that a negative slide is read with a parasitaemia above the LOB and is consequently considered as positive. The International Organization for Standardization (ISO) recommends for the determination of LOB that α is set to 5%. Therefore, LOB corresponds to the 95^th^ percentile of the parasitaemia distribution of negative samples (Figure 2). In case of a Gaussian distribution of the results

(1) $$\text{LOB}=\mu_{B}+1.645\cdot \sigma_{B}$$

where μ_B_ is the mean and σ_B_ the standard deviation of the parasitaemia of negative slides.

In the case of a non-Gaussian distribution, the 95^th^ percentile is determined non-parametrically based on the ordered values [15]. The results are ranked in ascending order and the LOB corresponds to the result at position $N_{B}\cdot \frac{95}{100}+0.5.$

### Determination of the limit of detection (LOD)

The LOD was obtained by repeated measurements of low parasitaemia TBS. If the distribution of the results is Gaussian then a defined percentage of the results will exceed the LOB but the rest will be below and, therefore, be considered as negative (See Figure 2). This error is called type II error or β. For this study β = α = 5% was allowed. The usual procedure to determine the LOD is to produce several low parasitaemia TBS (4 to 6) and to read them several times in order to create a series of measurements for each slide. For each series of measurements, the standard deviation σ_y_ is calculated.

(2) $$\text{LOD}=\text{LOB}+c_{\beta }\cdot \sigma_{\text{pooled}}$$

*σ*_*pooled*_ is the pooled standard deviation of the measurements from each series and corresponds to an estimate of the total standard deviation. *c*_*β*_ is a bias-corrected estimate of the 95^th^ percentile of the standard normal distribution. Furthermore *c*_*β*_ and *σ*_*pooled*_ can be expressed by

(3) $$\sigma_{\text{pooled}}=\sqrt{\frac{\left(n-1\right)\cdot \Sigma_{i=1}^{n}\sigma_{y}^{2}}{f}}$$

where n-1 represents the degree of freedom per series with n the number of measurement per series (or slides), *σ*_*y*_ is the per series standard deviation and *f* = *N*_*s*_ - *K* represents the degree of freedom with *Ns* the total number of measurements and *K* the number of series (or slides) and

(4) $$C_{\beta }=\frac{z_{\alpha }}{1-\frac{1}{4\cdot f}}$$

where *z*_*α*_ is the α-quantile of the standard normal distribution. If data are not normally distributed but follow a Poisson distribution, they can be transformed by the square root transformation yielding the transformed values [16].

(5) $$Y_{i}=\sqrt{X_{i}}$$

with X_i_ the read parasitaemia and Y_i_ the square root transformed parasitaemia. The LOD is then calculated with the transformed values and then back transformed to the original unit.

### PCR

The presence or absence of parasites in the samples as well as the *Plasmodium* species were confirmed by nested PCR as previously described [17].

## Results

### Calculation of the LOB

For the determination of LOB, 20 negatives slides were prepared from the malaria naïve individuals. Five random sequences were prepared using 20 negatives slides and 20 positive slides. After blinding each sequence was read once leading to 100 measurements. For the determination of the LOD, five random sequences of six low parasitaemia slides and two negative slides were prepared and read by six different microscopists. Therefore, 60 additional measurements of the negative slides were produced and added to the previous results leading to a total of *N*_*B*_ = 160 measurements. The results were not normally distributed. Therefore, the 95^th^ percentile was determined non-parametrically by the method of the ordered values [15]. The results were ordered from the lowest to the highest parasitaemia (Table 1) and the LOB corresponding to the 95^th^ percentile was estimated as the result at the position $N_{B}\cdot \frac{95}{100}+0.5=152.5$. For both methods, the results at position 152 and 153 are 0 parasites/μL, hence the LOB of TBS microscopy was 0 parasites/μL.

**Table 1:** Results of the negative slides for Lambaréné and WHO methods

	**Results (pf/μl)**	
**Rank**	**Lambaréné**	**WHO**
160	40	70
159	18	44
158	16	0
157	10	0
156	0	0
155	0	0
154	0	0
**153**	**0**	**0**
**152**	**0**	**0**
151	0	0
150	0	0

The results of the slides have been ordered from the lowest parasitaemia to the highest. Below the rank 150, the slides have been read as negative. The 95^th^ percentile of the distribution is between position 152 and 153 (bold).

### Calculation of the LOD

Several low parasitaemia TBS were prepared and six of them, representative of the different Giemsa stains and with a parasitaemia between 10 and 100 parasites/μL were selected, put in a random order together with two negative slides (see above) and blinded. After the first sequence was read by all microscopists, the slides were randomized and blinded again in order to create a new sequence. A total of five such sequences were prepared and read by six microscopists. Thus, at the end each slide was read five times giving a total of *N*_*S*_ = 6 slides × 5 sequences × 6 microscopists = 180 measurements of the low parasitaemia slides. The data were not normally distributed, so they were transformed by the square root transformation [16]. The values *Y*_*i*_ were obtained and used to calculate the pooled standard deviation according to formula (3) (see Table 2). For example for the Lambaréné method:

$$\begin{array}{ll} \;\sigma_{\text{pooled}} & =\sqrt{\frac{\left(n-1\right)\cdot \Sigma_{i=1}^{n}\sigma_{y}^{2}}{f}}\\ & =\sqrt{\frac{\left(30-1\right)\cdot \left(2.75^{2}+3.52^{2}+1.57^{2}+2.59^{2}+2.45^{2}+2.19^{2}\right)}{180-6}} \end{array}$$

$$\sigma_{\text{pooled}}=2.58$$

Because the transformed values *Y*_*i*_ were used for the calculation, *σ*_*pooled*_ has to be back-transformed by formula (6) [18].

(6) $$\sigma_{x}=2\cdot \sqrt{X}\cdot \sigma_{\text{pooled}}$$

Here *X* represents the mean of the measurements on the original scale. In the case of the Lambaréné method, *X =* 54 parasites/μL. So finally, $\sigma_{x}=2\cdot \sqrt{54}.2.58=37.9$. The LOD corresponds then to *LOD* = *LOB* + *c*_*β*_ ⋅ *σ*_*x*_.

$${\text{LOD}}_{\text{Lamba}}=0+1.647\cdot 37.9=62\;\text{parasites}/\mu L$$

The LOD for the WHO method (*LOD*_*WHO*_) was calculated in the same way and gave 88 parasites/μL.

**Table 2:** Results of each series of measurement of low parasitaemia slides prepared according to Lambaréné or WHO method before transformation (in parasites/μL) and after square root transformation

**Method: Lambaréné**	**Slide ID**					
	**LOD-L-21**	**LOD-L-16**	**LOD-L-20**	**LOD-L-17**	**LOD-L-18**	**LOD-L-12**
Non transformed						
Mean	105	77	63	45	26	8
Median	90	63	52	35	22	0
σ	60	59	27	34	20	11
Transformed						
Mean	9.9	8.1	7.8	6.2	4.5	1.9
Median	9.5	7.9	7.2	5.9	4.7	0
σ	2.75	3.52	1.57	2.59	2.45	2.19
**Method: WHO**	**Slide ID**					
	**LOD-W-21**	**LOD-W-18**	**LOD-W-19**	**LOD-W-20**	**LOD-W-17-4**	**LOD-W-17-1**
Non transformed						
Mean	116	45	102	65	35	27
Median	112	47	84	66	31	15
σ	35	41	56	40	36	43
Transformed						
Mean	10.6	5.3	9.7	7.5	4.5	3.5
Median	10.6	6.8	9.2	8.1	5.5	3.8
σ	1.73	4.11	2.87	2.94	3.87	3.83

Each slide was read 5 times by 6 different microscopists.

## PCR results

The negative samples were found negative by nested PCR. The low parasitaemia samples were positive for *Plasmodium falciparum,* but negative for *Plasmodium vivax*, *Plasmodium malariae* and *Plasmodium ovale*.

## Discussion

Accurate diagnosis is an important tool in the fight against malaria and the universal access to a parasitological test is part of WHO objectives [1]. Microscopy of TBS remains the gold standard despite the emergence of new diagnostic methods such as Rapid Diagnostic Test (RDT) or PCR. Surprisingly, only little information is available on the diagnostic performance of this method. Two important characteristics describing the performance of a laboratory method are the LOB and LOD. In the case of TBS, they depend on the quality of the sampling, the slide and the microscope, the number of examined HPFs as well as the expertise of the reader [19].

Adapting guidelines edited by the CLSI/NCCLS [14], a LOB of 0 parasites/μL was found, meaning that at least 95% of the time, a negative slide was correctly identified as negative. This shows a very good specificity of TBS in this particular setting which is concordant with other studies showing 99% specificity under optimal conditions [20]. The LODs for the Lambaréné and the WHO method were 62 parasites/μL and 88 parasites/μL respectively, showing that the Lambaréné method is slightly more sensitive than the WHO method in the location where it was invented. The interpretation of this finding is that a sample, with a parasitaemia above this limit, has a 95% probability to be correctly read as positive. However below this limit, the probability that the slide is mistaken as negative is higher than 5%. It is important to note that LOB/LOD studies are purely context dependent (e.g. in another setting the WHO method may result in better LOD) and shall be integrated in the routine laboratory work to avoid potential confounders such as reading under “competition”, which is likely to result in artificially low LODs.

Although no similar work could be found in the literature, the results are consistent with estimates of sensitivity in the field. Under optimal conditions sensitivity is approximately four parasites/μL but requires extended reading times: inspection of approximately 200 HPFs (the equivalent of approximately 0.5 μL of blood) is required to detect two unambiguous parasites (diagnosis is usually not based on detection of a single parasite), hence one parasite per 100 HPFs corresponds to a parasitaemia of approximately four parasites/μL [20,21]. In the field the sensitivity is estimated to be lower, commonly between 40–100 parasites/μL [22-24].

The most important discrimination of TBS is qualitative (negative *versus* positive) since it triggers anti-malarial chemotherapy. LOB and LOD estimates allow the reliable discrimination between positive and negative samples and consequently, they can help to improve patient care and to reduce the health care cost due to inadequate therapy, an issue that is particularly important in economically weak countries. A study from Kenya shows that microscopy diagnostics can generate cost savings but the effect would be more important if clinical practice were revised and notably if results were respected [25]. Parasite density is associated with disease severity [26] and it is also important in the setting of clinical trials with defined cut-offs for outcome definitions [27]. In endemic areas, distinguishing malaria from other diseases even when parasites are present is not straightforward because of the non-specific symptoms of the disease [28]. Therefore, a reliable parasite cut-off must be determined and standardization between readers and study sites must be achieved. This method provides a tool for quality control, which is easy to implement and allows comparison between readers and diagnostic units.

## Conclusion

Providing accurate and reliable data is of primary importance for every diagnostic laboratory therefore for most of the analytical methods and instruments, performance characteristics such as LOD and LOB are determined. This has not been done previously for TBS microscopy although this method has been used for more than one century. The LOB and LOD values determined in this study are specific for the setting under study, however the method is easily applicable in any laboratory performing TBS microscopy. It shall be used not only to provide information on the performance of a laboratory but also to improve clinicians’ trust into laboratory diagnosis of malaria.

## Abbreviations

HPF: High power field; LOB: Limit of blank; LOD: Limit of Detection; σ: Standard deviation; TBS: Thick blood smear.

## Competing interests

The authors declare that they have no competing interests.

## Authors’ contribution

BM and BL proposed and helped to design the study and corrected the manuscript; SJZL designed and performed the study; FJ helped to design and to perform the study and drafted the manuscript, TE supervised statistical analysis and corrected the manuscript. All authors read and approved the final manuscript.
